# Emotional and Behavioral Changes in Older Adults With High Risk of Cognitive Impairment During the COVID-19 Pandemic

**DOI:** 10.3389/fpsyg.2021.719774

**Published:** 2021-09-30

**Authors:** Jiangning Fu, Xiaomei Liu, Jing Li, Zhuoya Ma, Juan Li

**Affiliations:** ^1^CAS Key Laboratory of Mental Health, Center on Aging Psychology, Institute of Psychology, Chinese Academy of Sciences, Beijing, China; ^2^Department of Psychology, University of Chinese Academy of Sciences, Beijing, China; ^3^Magnetic Resonance Imaging Research Center, Institute of Psychology, Chinese Academy of Sciences, Beijing, China

**Keywords:** older adults, high risk of cognitive impairment, COVID-19, emotional states, protective behaviors, mental health

## Abstract

COVID-19 is not only a threat to physical health but also a stressor to mental health, particularly for older adults. Previous studies have indicated that healthy older adults have resilience to cope with such stressful event through emotional and behavioral effort. However, very few have investigated the coping ability of older adults with High Risk of Cognitive Impairment (HRCI), as they are characterized with risk factors that can make them more vulnerable to COVID-19 in both physical and mental aspects. To examine whether older adults with HRCI were able to cope with and recover from the outbreak of COVID-19, we investigated the changes of their self-reported emotional states and intentions of taking protective behaviors between the outbreak period (data collected from February 17th to 24th, 2020) and the remission period (data collected from April 7th to 20th, 2020). The results showed that compared with the outbreak period, older adults with HRCI showed better emotional states and higher levels of intention to take more protective behaviors during the remission period. Subgroup analysis showed that even those who showed relatively poor coping abilities during the outbreak period could gradually improve their emotional states and intend to take more protective behaviors later on in the remission period. Therefore, these results suggested that older adults with HRCI were able to cope with and recover from the pandemic outbreak.

## Introduction

The rapid spread of COVID-19 pandemic has posed a huge threat to physical health. At the same time, it also creates drastic negative effects on mental health as a result of worries about contagion, fears of shortages, and restricted social contact. The pandemic impacts the entire population, but older adults are more prone to COVID-19 with higher case-fatality rate compared to other age groups (Wu and McGoogan, 2020). As the pandemic could be more challenging for older adults to cope with, a few studies have found that healthy older adults have resilience when facing the pandemic, shown by having relatively better emotional states compared to younger adults (Benke et al., 2020; Carstensen et al., 2020; Klaiber et al., 2021). However, very few have looked at the coping ability of older adults with High Risk of Cognitive Impairment (HRCI), as they are characterized with risk factors that can make them more vulnerable to COVID-19 both physically and mentally. Therefore, this research aimed to investigate the coping ability of older adults with HRCI by examining the changes of their emotional states and intentions of taking protective behaviors from the outbreak period to the remission period of COVID-19 in 2020.

Coping is defined as managing stressors through one’s cognitive and behavioral efforts (Folkman, 1984). There are usually two types of coping strategies, based on the functions: emotion-focused strategies, which refer to managing the emotions that are associated with the stressor; and problem-focused strategies, which refer to taking actions to deal with the problem directly (Folkman, 1984; Lazarus and Folkman, 1984). Specifically, to the interests of this study, emotional states and behavioral intentions to take protective behaviors were used to measure the coping ability of HRCI older adults during the pandemic.

Successful coping is very important to people’s physical health, mental health, and quality of life during the pandemic (Shamblaw et al., 2021). Some studies have demonstrated that older adults tend to show better resilience compared to younger adults. For instance, Carstensen et al. (2020) surveyed nearly 1,000 Americans (aged 18–76years old) online about their positive and negative emotional experiences when COVID-19 started to surge in the United States in April 2020. The results indicated that age was positively associated with an increase in the frequency and intensity of positive emotions and a reduction in frequency and intensity of negative emotions. Benke et al. (2020) found similar age differences with a German sample, such that older adults were less likely to show depression and anxiety than younger adults during the stay-at-home orders. Furthermore, Klaiber et al. (2021) showed that while older and younger adults did not differ in their exposure to COVID-19 stressors, older adults had better emotional states compared to their younger counterparts.

These findings can be explained by Socioemotional Selectivity Theory (SST; Carstensen et al., 1999), which posits a developmental shift in motivation of setting goals for information-seeking vs. emotional well-being. SST suggests that as people age, they perceive time left as more limited, which would lead to prioritizing emotionally meaningful goals rather than information-seeking goals. As a result, the emotional well-being would be preserved or even enhanced with aging. Furthermore, the Strength and Vulnerability Integration (SAVI) model purposed by (Charles, 2010) incorporates the perceived time left as a mechanism, posited by SST theory, but also posits that time lived is an important mechanism whereby people gain experience and knowledge from navigating and solving problems of daily life. The SAVI model emphasizes that the enhancement of emotional well-being with aging is due to older adults’ better ability of emotional regulation to avoid or limit the elicitation of negative emotions. Taking these theoretical accounts together, the SST/SAVI model suggests that older adults are able to cope with the stress in daily life.

However, it is important to note that the SST/SAVI model does not discuss the influence of personal characteristics on older adults’ coping ability. As a matter of fact, according to the definition of coping (Folkman, 1984), which involves cognitive efforts, it is reasonable to suspect that older adults who are more prone to cognitive declines might have difficulties in coping with stressors, compared to their healthy counterparts. Indeed, some researchers have found that older adults diagnosed with cognitive impairment, for instance Alzheimer’s Disease (AD) patients, had worse resilience, shown by lower levels of emotional well-being and orientation towards problem-focused coping strategies, compared to healthy older adults (Meléndez et al., 2018).

There has not been a lot of research done on the coping ability of older adults with HRCI during the pandemic. We believe research on this group of older adults is very worth noting, because HRCI individuals are characterized with risk factors that can limit their coping abilities and make them physically vulnerable to the pandemic. At the same time, they tend to receive less care and attention compared to those who are already diagnosed with cognitive impairment (e.g., AD patients).

Older adults with HRCI are characterized as having subjective memory complaints (Gallassi et al., 2010), slightly lower cognitive performance than healthy aging population (Amieva et al., 2005), risk genes, such as Apoe ε4 (Hsiung et al., 2004), poor emotional state (Weisenbach et al., 2012), low education attainment (Beydoun et al., 2014), and cardiovascular risk factors, such as lack of physical activities, smoking, diabetes, heart disease, hypertension (Baumgart et al., 2015). It is critical to note that these risk factors overlap with those of COVID-19. For example, having cardiovascular disease was associated with a higher mortality rate among COVID-19 patients (Li X. et al., 2020). Being a smoker and having pre-existing conditions, such as hypertension and diabetes, also greatly influenced the prognosis of COVID-19 (Zheng et al., 2020). Therefore, the pandemic may become a more stressful event for older adults with HRCI to cope with as they are more vulnerable to the disease.

The coping ability may be impeded for older adults with HRCI, as they already show certain degree of cognitive decline or have a high risk of cognitive decline (i.e., Apoe ε4 carriers; Reitz and Mayeux, 2010). According to the transactional theory of stress and coping (Lazarus and Folkman, 1984), when an individual encounters a stressful event, before any coping strategies are taken, he/she needs to appraise the stressor and determine how to deal with this situation. This appraisal process is a cognitive process that relies on cognitive abilities (Lazarus and Folkman, 1984; Kim and Duda, 2003). As HRCI older adults are at risk of cognitive decline, they may be unable to effectively use coping strategies when facing stressors.

There are other demographic and social factors that can influence coping abilities. Previous studies have found that there are gender differences, such that women are more likely to use the emotion-based response as coping strategies (Ptacek et al., 1992). Some studies also indicated that higher education level is related to better coping ability (Cui et al., 2019). In addition, social support is also an important factor, as it can improve the coping ability of older adults to support emotional well-being (Strine et al., 2008; Li et al., 2014). Social support is part of psychological resources, and low-level psychological resources are associated with low mental health levels (Greenglass, 2002) and worse daily life function (Kempen et al., 1999), which are highly related to older adults’ coping ability (Greenglass et al., 2006).

Therefore, this current study aimed to investigate whether older adults with HRCI living in communities in Beijing, China, were able to cope with the COVID-19 pandemic. Due to the severity of the pandemic varying over time, we selected two periods, which were the outbreak period and the remission period, so that we could examine the changes of emotional states and intentions of taking protective behaviors. During the outbreak period, the number of contracted cases grew exponentially and the rate of growing reached its peak level, which could be considered the most severe period that put drastic stress on people daily life. Whereas, during the remission period, as the situation became more controlled and people learned more about the disease, improvement in emotional states and taking protective behaviors should be observed. However, as discussed above, it may be very difficult for older adults with HRCI to cope with the pandemic due to their physical and mental vulnerability. It was predicted that in general, older adults with HRCI may not be able to improve their emotional states and take more protective measures against the disease in remission period compared to the outbreak period. In addition, since there might be individual differences in coping abilities, we further conducted subgroup analysis to first identify those who showed relatively good resilience at the beginning of the pandemic (i.e., individuals who had relatively better emotional states and already intended to take almost all protective behaviors at outbreak period) from those who did not. Then, we compared the subgroups in terms of the changes of their coping strategies from the outbreak period to the remission period.

## Materials and Methods

### Participants

A total of 158 participants were recruited from an ongoing intervention (Comprehensive intervention of cognition, emotion, and nutrition for older adults with HRCI) by convenient sampling. Two additional participants were excluded from analysis as they did not complete the study. All participants were from communities in Chaoyang District of Beijing. Based on previous studies, the current study followed the inclusion criteria of the ongoing intervention to identify older adults with HRCI^1^: (1) age≥60years; (2) score of Dementia Screening Interview (Galvin et al., 2005)≥1; (3) more than one risk factor of having education level below the primary school, lacking physical exercise, having depression score≥16 (Center for Epidemiologic Studies Depression Scale, CES-D; Radloff, 1977), smoking, or having hypertension, diabetes, heart disease, or cerebral infarction; (4) Apoe ε4 carrier, or cognitive performance score (measured by the Mini-Mental State Examination, MMSE; Folstein et al., 1975)<27 (the average of all participants in the ongoing intervention), or Associative Memory Test score≤6.5. Individuals with severe cognitive declines (MMSE<18), extremely high levels of depressive symptoms (CES-D>28), or hearing impairment were excluded, as they did not fit the criteria of older adults with HRCI or had troubles completing the interviews over phone.

### Design and Procedure

A two-wave fixed cohort study design was adopted to assess the emotional states and the actions of taking protective behaviors in older adults with HRCI. The study protocol was approved by Ethics Committee of the Institute of Psychology, the Chinese Academy of Sciences, and the ethic code was H20012.

**Figure 1:**
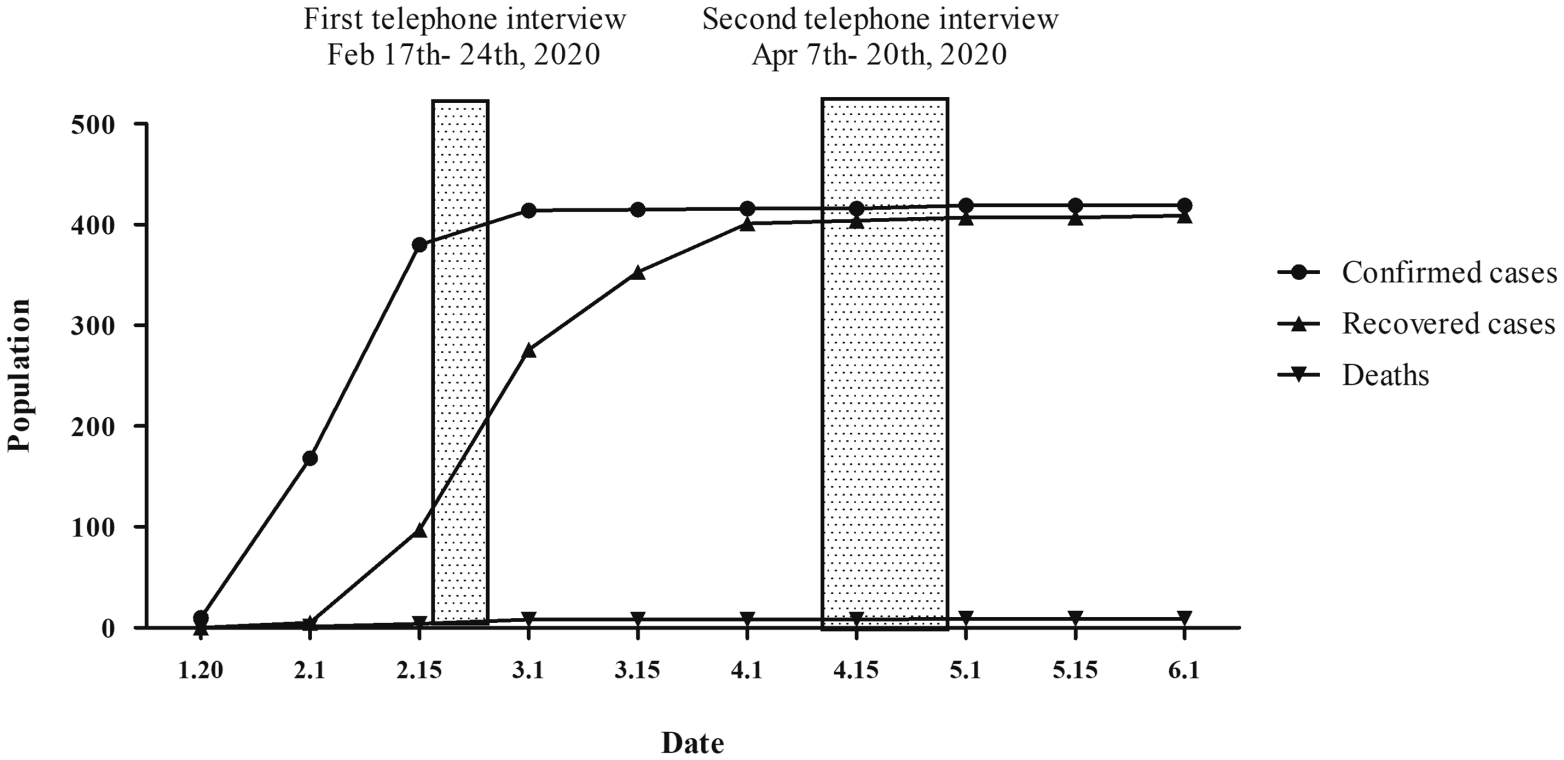
The epidemic trend of the 2019 coronavirus disease (COVID-19) in Beijing from January 10th to June 1st 2020.

As shown in Figure 1, according to the official report of the Beijing Municipal Health Commission, from January to March 4, 2020, the number of locally confirmed cases in Beijing continued to rise and the rate of daily confirmed cases reached its peak level, so this period was regarded as an outbreak period. Since then, although there were sporadic confirmed cases in Beijing, as of April 7, local cases in Beijing had not increased for 14 consecutive days. At the same time, the World Health Organization announced that COVID-19 had entered a remission period in China (K. Zhang et al., 2021). Thus, the period starting from April was called the remission period in the current study.

Two telephone interviews were conducted with each participant. One was during the outbreak period, from February 17th to 24th, 2020, and the other was during the remission period, from April 7th to 20th, 2020. As all the dwellers in Beijing were asked to follow the strict stay-at-home order to minimize face-to-face interactions, interviews were delivered through telephone by trained personnel during daytime. Interviewers were asked to read a formatted script when asking questions from the questionnaires. Also, simple sentences were used when communicating with participants to make sure that they could understand all of the questions. Considering the poor concentration and physical condition of the older adults with HRCI, each interview was completed within 10min. Informed consent was obtained verbally at the beginning of the first interview. Participants were asked about their sources of social support, satisfaction of the whole society in combating the COVID-19 pandemic, emotional states, and intention to take protective behaviors against the pandemic. The measures were identical across the two interviews.

### Measures

#### Demographics and Cognitive Function

Participants’ demographic and cognitive function information was obtained from the ongoing intervention mentioned earlier. Demographic information included gender, age, and years of education. Information on cognitive function included MMSE scores and Apoe ε4 gene carrying status, coded as “1” for carrier and “0” for non-carrier (Davies et al., 2015).

#### Sources of Social Support and Satisfaction

The sources of social support were measured by a question asking “Whom do you keep in touch with every day, family member, community workers, or none?” Responses were “2” for receiving support from both family and community, “1” for receiving support only from family or community, or “0” for receiving no support. This variable was treated as a continuous variable in subsequent analyses. Participants were also asked to rate how they were satisfied with the whole society in combating the COVID-19 pandemic, using a 5-point Likert scale from extremely unsatisfied to very satisfied.

#### Emotional States

As shown in Table 1, a self-designed scale of negative emotional status was used to assess feelings of anxiety, depression, anger, and pessimism. Catastrophic events may cause a variety of negative emotional responses in older adults. Since depression, anxiety, and anger are the most representative negative emotions that have been reported by COVID-19 related research (Li S. et al., 2020), we included one question for each of those emotions in the scale. In addition, studies have shown that feelings of pessimism had a negative effect on the psychological resilience in older adults (Almazan et al., 2018), thus, a question about pessimism was also included in the scale. Participants rated their feelings of anxiety, anger, depression, and pessimism, on a 5-point scale, ranging from “1” (strongly disagree) to “5” (strongly agree). The scale had good internal consistency (Cronbach’s ɑ =0.721). Unidimensionality of the scale was further verified with a confirmatory factor analysis (CFA). The result showed a good fit (CFI=0.999, TLI=0.998, RMSEA=0.017; Huang et al., 2008). Thus, the scale score was treated as a total score of all four questions, ranging from 5 to 20, with a higher score indicating worse or more negative emotional states.

**Table 1:** Items for the emotional states scale and protective scale.

Measure	Instructions	Scale	Item
Emotional states	Please choose an option according to your emotional state of this week:	1 (strongly disagree) to 5 (strongly agree)	1.I’m more nervous and anxious than usual.
			2.I’m angry at the negative news during the epidemic.
			3.I feel depressed.
			4.I am pessimistic about the future.
Protective behaviors	What changes will you make after the outbreak:	Yes or No	1.A more regular lifestyle.
			2.To do more exercise.
			3.To be more aware of the importance of family.
			4.To pay more attention to distinguishing news on the Internet.
			5.To pay more attention to personal hygiene.
			6.Respond more to the government’s requirements (not to leave the city, quarantine at home during the pandemic, etc.).

#### Protective Behaviors

As demonstrated in Table 1, a self-compiled scale was developed to investigate what behaviors people intended to take to protect themselves against the pandemic, including physical health (regular life, physical exercise, personal hygiene, and compliance with public order) and mental health (attitude towards family, identification of true and false information from the internet). This scale included three aspects. One was the positive changes in daily lifestyle, including regular life and more physical exercise. The second aspect was the effort to reduce negative emotions. Previous studies have shown that the lack of family support may lead to depression in older adults (Chou et al., 2006), and paying attention to unofficial and negative false news may lead to panic and negative emotions (Chao et al., 2020). Thus, seeking family support and effectively identifying true and false information on the Internet were included. The third aspect was to take measures to protect one’s self from the virus, including paying more attention to personal hygiene and complying with the stay-at-home order. As a result, the scale had six items, with two items from each aspect, to assess the behavioral effort in coping with the pandemic. Participants reported their answers using a dichotomous option (yes=1/no=0), and the scale internal consistency was good (Cronbach’s ɑ=0.735). To confirm the factor structure, a CFA for the one factor model was conducted, which indicated acceptable fit (CFI=0.951, TLI=0.918, RMSEA=0.085; Huang et al., 2008). Total score was used, with a higher value indicating more positive behaviors the participants tended to take.

### Statistical Analysis

Chi-square test, paired-samples t-test, and Wilcoxon rank-sum test were performed to examine the changes in emotional states and actions of taking protective behaviors between the outbreak and remission periods. All tests were two-tailed, with a significance level of *p*<0.05. CFA for confirming the unidimensionality of the emotional states scale and protective behavior scale was conducted with Mplus software (version 7.0). Unless specified, all other analyses were conducted using the IBM SPSS Statistics software (version 22).

## Results

### Participant Characteristics

Table 2 presents the participant characteristics, including information on demographics and general cognitive performance measured by MMSE obtained from the ongoing intervention project, as well as information on the sources of social support and the personal satisfaction of how the whole society had combated the COVID-19 at the outbreak and remission periods. The sources of social support showed a significant difference between the two periods. Specifically, the proportion of HRCI older adults receiving both family and community support was higher at the outbreak period relative to the remission period; whereas, the proportion of participants receiving only type of support, either from family or community, increased from the outbreak period to the remission period. For the satisfaction of how the whole society had combated the COVID-19, overall, participants were pretty satisfied at the two period of the pandemic, with scores higher than the neutral level (value of 3 on a 5-point Likert scale).^2^

**Table 2:** Participant characteristics at outbreak and remission periods of COVID-19 pandemic (*N*=158).

	Outbreak period	Remission period	*t/χ* ^2^	*Value of p*
	N (%) or Mean (SD)	N (%) or Mean (SD)		
**Gender**				
Male	50 (31.6)	—	—	—
Female	108 (68.4)			
Age	71.24 (6.03)	—	—	—
Education years	8.55 (3.53)	—	—	—
**Apoe ε4 Carrie**r				
Yes	37 (23.4)	—	—	—
No	121 (76.6)			
MMSE	25.93 (2.61)	—	—	—
**Social support**				
Family and Community	44 (27.8)	20 (12.7)	11.684	0.003
Community or Family	108 (68.4)	133 (84.2)		
None	6 (3.8)	5 (3.2)		
Satisfaction to combating COVID-19	4.24 (0.89)	3.78 (1.50)	3.410	0.001

### Changes in Emotional States and Protective Behaviors

Table 3 presents the changes of emotional states and actions of taking protective behaviors between the outbreak and remission periods. Overall, older adults with HRCI showed less negative emotional states in the remission period relative to the outbreak period, which was indicated by a reduction in the total score of negative emotions. For the behavioral intentions of taking protective behaviors, older adults with HRCI reported a higher level of tendency to take more protective behaviors at the remission period compared to the outbreak period. Specifically, participants were more likely to pay attention to personal hygiene (i.e., Item 5) in the remission period. In addition, there was also a numerical trend to take more behaviors about having a more regular lifestyle (i.e., Item 1) and being more aware of the importance of family (i.e., Item 3) in the remission period.

**Table 3:** Changes of emotional states and protective behaviors between two outbreak and remission periods of COVID-19 pandemic (*N*=158).

Measure	Outbreak period Mean (SE) or N (%)	Remission period Mean (SE) or N (%)	Statistics	*Value of p*
Emotional scores	9.17 (0.29)	7.85 (0.23)	4.687^b^	<0.001
**Protective behaviors**				
Item 1	95 (60.1)	111 (70.3)	3.570^a^	0.059
Item 2	129 (81.6)	130 (82.3)	0.021^a^	0.884
Item 3	117 (74.1)	130 (82.3)	3.133^a^	0.077
Item 4	91 (57.6)	95 (60.1)	0.209^a^	0.647
Item 5	131 (82.9)	144 (91.1)	4.736^a^	0.030
Item 6	143 (90.5)	141 (89.2)	0.139^a^	0.709
Item Total	706 (74.5)	751 (79.2)	-2.100^c^	0.036

a *chi-square test*.

b *paired samples t-test*.

c *Wilcoxon rank sum test*.

### Subgroup Analysis

To identify the individuals who had relatively better emotional states or already intended to take almost all protective behaviors from those who did not at the outbreak period, we performed latent profile analysis (LPA) and latent class analysis (LCA; Gibson, 1959). LPA was conducted to identify the emotion subgroups, as the scores from the emotional states scale were treated as continuous variables. LCA was conducted to identify the behavior subgroups, since the item scores from the protective behavior scale were treated as dichotomous responses. LPA and LCA were conducted with Mplus software (version 7.0). Models with two through four profiles were estimated. Best model fit was assessed using sample size-adjusted Bayesian information criteria (aBIC; Yang, 2006), where the smallest number indicates the best fit. The number of profiles was determined based on the Lo–Mendell–Rubin likelihood Ratio Test (LMRL; Lo et al., 2001), which tests the significance of the−2 log-likelihood difference between models with k and k-1 profiles, and the Bootstrap Likelihood Ratio Test (BLRT; Nylund et al., 2007).

LPA was computed using all 158 participants who completed the emotional states scale. Model fit (aBIC) for the current sample was 2026.804 for one profile, 1838.092 for two profiles, 1797.872 for three profiles, and 1612.923 for four profiles. The LMRL showed that the two-profile model was significantly better than the one-profile model (*p*<0.001); the three-profile model was significantly better than the two-profile model (*p*<0.05); but there were no differences between the four-profile and the three-profile models (*p*>0.05). The BLRT of all profiles were significant (*p*<0.001). For the aBIC, the value decreased as the number of profiles increased in the model, and there was an obvious inflection point at the two-profile model. Therefore, we considered that both two-profile and three-profile were acceptable models (Petras and Masyn, 2010). Because our goal was to separate the participants with good emotional coping from those with poor coping abilities, and also to ensure an adequate number of individuals in each subgroup, the two-profile model was finally adopted as the grouping method. The “Good Emotional Coping (GEC)” group (*n*=98, 62.0% of participants) showed low average scores at all four items, the “Poor Emotional Coping (PEC)” profile (*n*=60, 38.0% of participants) showed high average scores at all four items. In fact, the two groups had distinct score distributions at all of the four items. As shown in Table 4, the GEC group had fewer negative emotions at the outbreak period compared to the PEC group. In terms of the group differences in the participant characteristics, the PEC group had a larger proportion of females than males, compared to the GEC group. Other participant characteristics did not vary between the two groups.

**Table 4:** Participant characteristics of Good Emotional Coping (GEC) group and Poor Emotional Coping (PEC) group at the outbreak period.

	GEC Group	PEC Group	*t/χ* ^2^	*Value of p*
	(*n*=98)	(*n*=60)		
	Mean (SD) or N (%)	Mean (SD) or N (%)		
Emotional states in outbreak period	6.92 (2.47)	12.68 (2.13)	−14.976	<0.001^**^
**Gender**				
Male	38 (38.8)	12 (20.0)	6.065	0.014^*^
Female	60 (61.2)	48 (80.0)		
Age (Yrs)	71.95 (5.9)	70.08 (6.02)	1.902	0.059
Education years	8.58 (3.57)	8.49 (3.49)	0.155	0.877
**Apoe ε4 Carrier**				
Yes	121 (21.4)	16 (26.7)	0.569	0.451
No	77 (78.6)	44 (73.3)		
MMSE	26.08 (2.40)	25.68 (2.93)	0.929	0.354
**Social support**				
Family and Community	25 (25.5)	19 (31.7)	1.703	0.427
Community or Family	68 (69.4)	40 (66.7)		
None	5 (5.1)	1 (1.7)		

* *p< 0.05*;

** *p< 0.01*.

**Figure 2:**
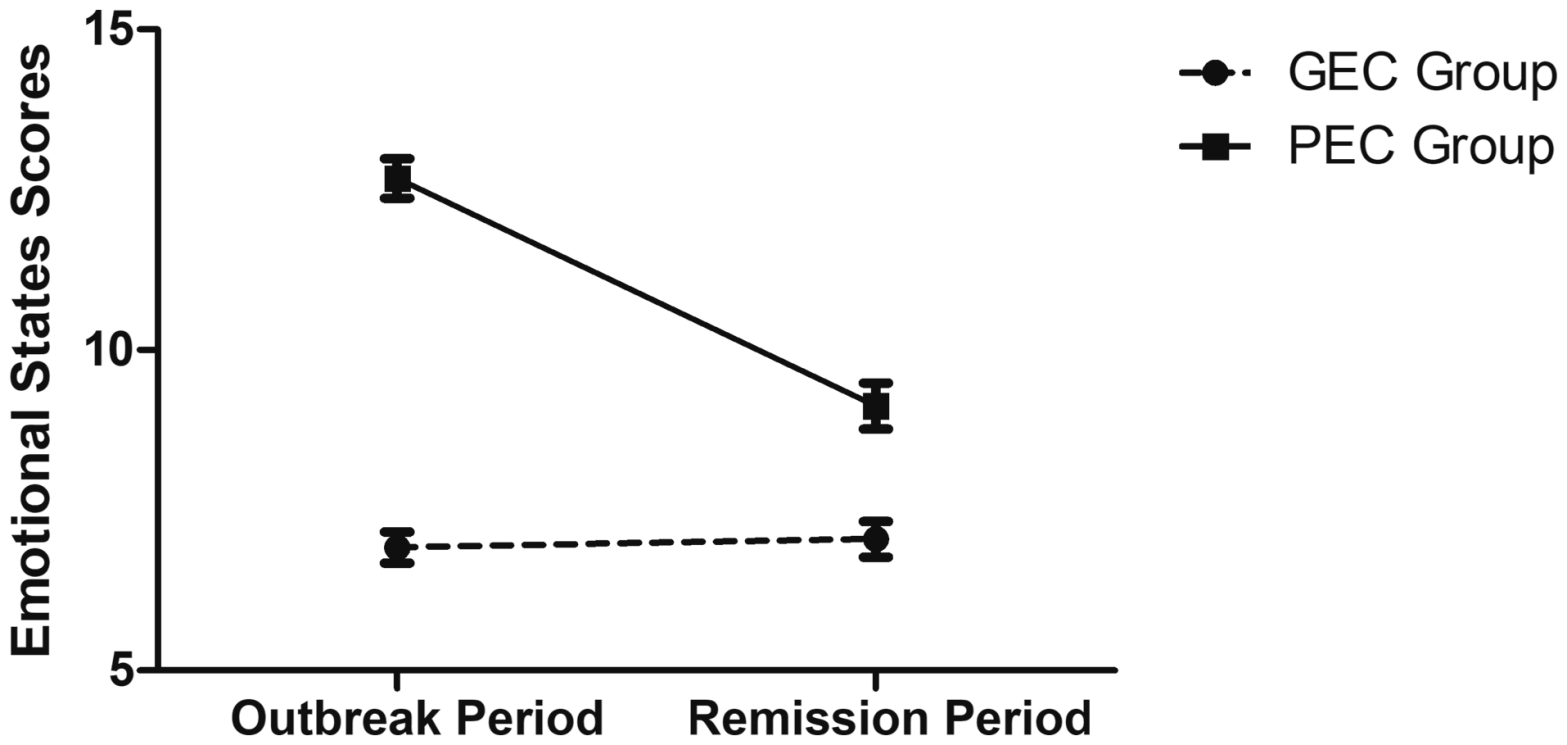
Scores of emotional states in the outbreak and remission periods for the Good Emotional Coping (GEC) group and the Poor Emotional Coping (PEC) group. Error bar presents standard error of the mean.

A mixed model analysis of variance (ANOVA) was conducted to investigate the changes of emotional states between the two subgroups, where Group (GEC vs. PEC) was the between-subject variable, Period (Outbreak vs. Remission) was the within-subject variable. Since there were only gender differences between the two groups at the outbreak period, gender was controlled as a covariate. As shown in Figure 2, there was a significant interaction between Group and Period, *F*(1, 155)=50.149, *p*<0.001, $\eta_{p}{}^{2}$=0.244. *Post hoc* analysis revealed that the GEC group showed no significant difference between the outbreak period and remission period, *p*=0.658. Meanwhile, there was a significant and obvious reduction in negative emotions for the PEC group from the outbreak period to the remission period, *p*<0.001. The main effect of Period was significant, *F*(1, 155)=32.088, *p*<0.001, $\eta_{p}{}^{2}$=0.172, which was consistent with the findings in Table 3. Other effects were not significant, *F*’s<1.

LCA was conducted to identify subgroups from the 158 participants who completed the protective behavior scale. The results indicated that the best-fitting model was the two-class model, which had the lowest aBIC (1014.498 for one-class model, 878.930 for two-class model, and 878.989 for three-class model). BLRT and LMR results also indicated that the two-class model was significantly better than the one-class model, *p*’s<0.001; whereas the three-class model was not significantly different from the two-class model, *p*’s>0.1. According to the response characteristics on the 6 items for the two-class model, 72.5% of participants (*n*=116) were classified as the Good Behavioral Coping (GBC) group, with a high proportion of taking more protective behaviors at the outbreak period. About 27.5% of the participants (*n*=42) were classified as the Poor Behavioral Coping (PBC) group, with a low proportion of taking protective behaviors at the outbreak period. As shown in Table 5, the GBC group reported intending to take more protective behaviors at the outbreak period compared to the PBC group. In terms of the group differences in participant characteristics, there was a significant difference in the sources of social support, such that the GBC group reported having more sources of social support (whether single source or dual source) compared to the PBC group at the outbreak period. Other participant characteristics did not vary between the two groups.

**Table 5:** Participant characteristics of the Good Behavioral Coping (GBC) group and Poor Behavioral Coping (PBC) group at the outbreak period.

	GBC Group	PBC Group	*t/χ* ^2^	*Value of p*
	(*n*=116)	(*n*=42)		
	Mean (SD) or N (%)	Mean (SD) or N (%)		
Protective behaviors in outbreak period	5.32 (0.79)	2.12 (1.02)	20.822	<0.001^**^
**Gender**				
Male	37 (31.9)	13 (31.0)	0.013	0.910
Female	79 (68.1)	29 (69.0)		
Age (Yrs)	71.00 (5.76)	71.93 (6.76)	−0.862	0.390
Education years	8.64 (3.44)	8.29 (3.78)	0.560	0.576
**Apoe ε4 Carrier**				
Yes	29 (25.0)	8 (19.0)	0.609	0.435
No	87 (75.0)	34 (81.0)		
MMSE	25.98 (2.59)	25.79 (2.71)	0.418	0.677
**Social support**				
Family and Community	41 (35.3)	3 (7.1)	13.015	0.001^**^
Community or Family	72 (62.1)	36 (85.7)		
None	3 (2.6)	3 (7.1)		

** *p< 0.01*.

**Figure 3:**
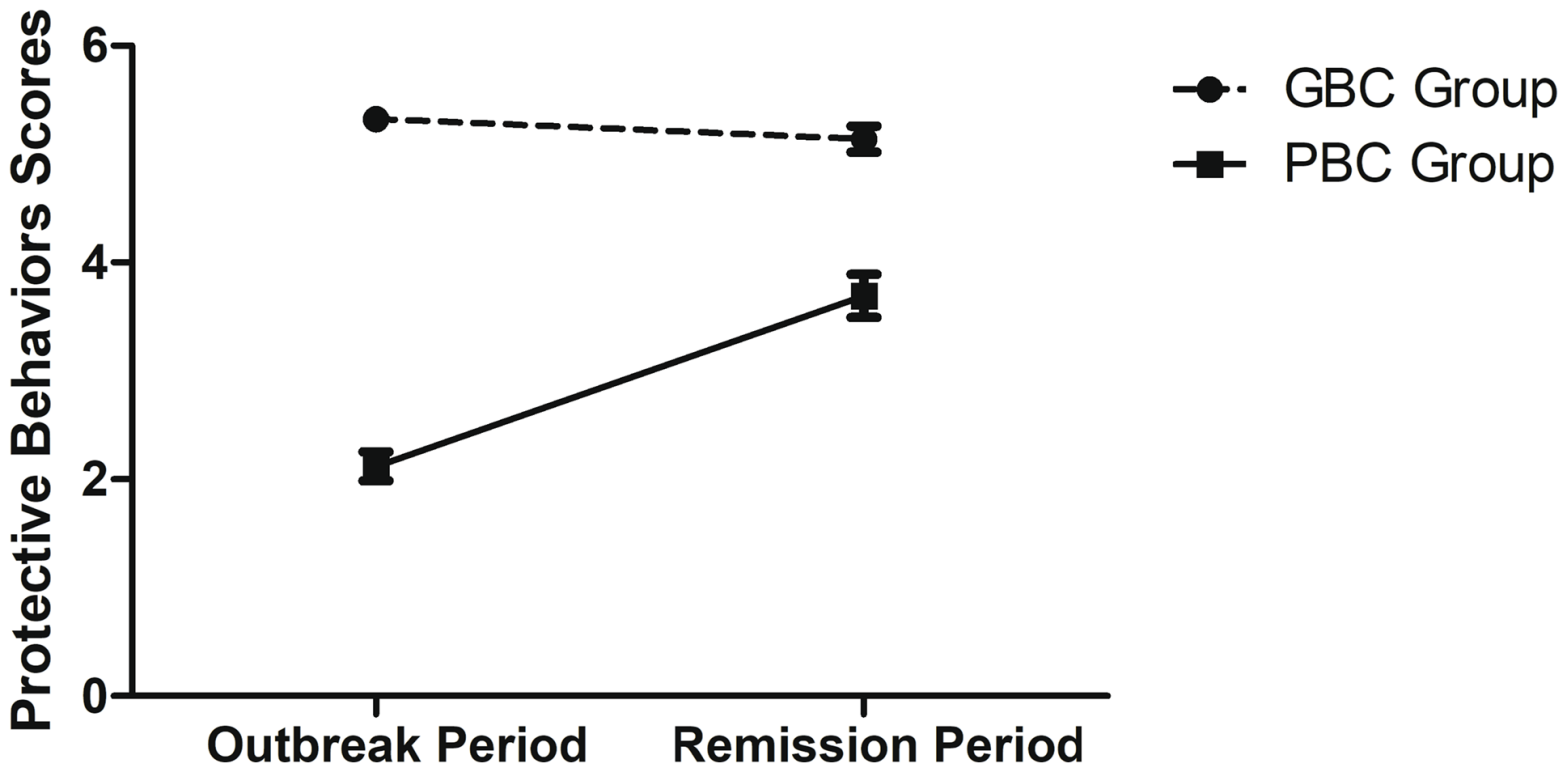
Scores of protective behaviors in the outbreak and remission periods for the Good Behavioral Coping (GBC) group and the Poor Behavioral Coping (PBC) group. Error bar presents standard error of the mean.

Similar to the analysis on changes of emotional states, a mixed model ANOVA was conducted to investigate the changes in the number of taking protective behaviors between the two subgroups between the outbreak and the remission periods. Since there were group differences at the outbreak period, the sources of social support were controlled as covariates. As shown in Figure 3, there was a significant interaction between Group and Period, *F*(1, 155)=45.170, *p*<0.001, $\eta_{p}{}^{2}$=0.226. *Post hoc* analysis revealed that the GBC group showed no difference between the two periods, *p*>0.07. However, for the PBC group, there was a significant increase in the number of reporting taking protective behaviors from the outbreak period to the remission period, *p*<0.001. Other effects were not significant, *F*’s<1, *p*>0.1.

## Discussion

Using telephone interviews, this study investigated the changes of emotional states and the actions of taking protective behaviors of older adults with HRCI between the outbreak and remission periods of COVID-19 pandemic. Overall, results showed that HRCI older adults were able to cope with the pandemic, as they showed fewer negative emotions and a tendency to take more protective behaviors from the outbreak to the remission period. Even those who showed relatively poor coping abilities at the beginning (i.e., at the outbreak period) can gradually improve their emotional states and adopt more protective behaviors later on during the remission period. This study was the first to examine the coping ability of older adults with HRCI during the COVID-19 pandemic, which could provide a valuable reference for practice.

Surprisingly, our hypothesis was not supported, since the findings showed that older adults with HRCI were able to demonstrate resilience during the pandemic. Previous studies indicated that older adults with risk of cognitive impairment used less emotional and supportive coping strategies compared with the population sample (Axelman et al., 2003), and the pathology of cognitive decline could hinder the effective adoption of coping strategies (Meléndez et al., 2018). Although our findings could not directly speak against those, HRCI older adults in this study did show coping abilities with improved emotional states and higher intentions of taking protective behaviors during the pandemic. It might be possible that although older adults with HRCI could have poor cognitive abilities, their motivation to orient goals to achieve emotional well-being remained preserved, so that they could still deal with stressors. This idea may be supported by the SST/SAVI model that emotionally meaningful goals would be prioritized among older adults, and the longer time lived would allow them to gain experience about how to disengage or de-escalate negative experiences.

Importantly, subgroup analysis results showed that although there were HRCI older adults that relatively did not cope well (i.e., those who had relatively more negative emotions or those who did not take enough protective behaviors) at the early stage of the pandemic, they were able to show improvements in the emotional and behavioral coping later on in the remission (*cf*. Figures 2, 3). Comparing these results to previous studies that showed healthy older adults had good resilience (e.g., Carstensen et al., 2020), it is possible that HRCI older adults might simply need more time to cope with stressors.

There were some individual differences between those who showed relatively good resilience at the beginning of the pandemic (i.e., individuals who had relatively better emotional states and had already taken all protective behaviors at outbreak period) from those who did not. In terms of emotional states, females tended to show more negative emotions than males, which may be related to the gender characteristics of coping strategies. When facing stressful events, women are more likely to make emotional reactions, probably due to the gender differences in socialization, such that women tend to seek more emotional support, and the differences in the stress situation that men and women usually encounter (Ptacek et al., 1992). In addition, since both community and family support can contribute to better mental health for older adults (Shen and Yeatts, 2013), receiving both sources could help HRCI older adults cope with stressors more effectively. These individual differences in coping abilities implied that when providing psychological assistance to older adults with HRCI, more attention should be given to females, and those who had fewer social support sources.

This study has some limitations. First, we did not recruit healthy older adults as a control group. Due to the pandemic outbreak, people were asked to comply with the strict stay-at-home order. Thus, we were unable to conduct face-to-face cognitive tests to recruit healthy older adults without HRCI as a control group. Future studies may further investigate the differences of coping abilities between healthy older adults and HRCI older adults when they respond to stressful events. In addition, considering the poor concentration and physical condition of older adults with HRCI, we were limited to perform a short interview with our participants rather than a comprehensive interview. As a result, we were limited to not being able to have a detailed examination of individual differences. For instance, when measuring social support, only the sources but not the amount of it was measured. However, as regular social contact was restricted during the pandemic, the amount of social support could dramatically decrease to become a flooring effect. On the other hand, the community, as the smallest administrative agency of the government, tended to have more interactions with the residents to provide assistance and support during the pandemic (Zhang et al., 2020). As a result, the sources of social support, such as from family and community, may play a more important role rather than the amount of social support during the pandemic (Shen and Yeatts, 2013). Future studies could include more items for social support scale in order to investigate it more accurately and comprehensively. Another issue was that this study did not find any difference in cognitive performance level between HRCI older adults who relatively good coping abilities at the most stressful time point of the pandemic (i.e., outbreak period) and those who had poor coping abilities. This result might suggest that the influence of cognitive function on the coping ability of older adults with HRCI may be too weak to be observed. Future studies may be conducted to include older adults with mild cognitive impairment or AD patients to further examine how different levels of cognitive impairment can influence the emotional and behavioral coping abilities.

## Conclusion

The present findings showed that older adults with HRCI had resilience to cope with the outbreak of COVID-19 in terms of improving their emotional states and taking protective behaviors. However, there are some individual differences that can impact the coping abilities, thus, certain subgroups of older adults with HRCI need to be paid with more attention when helping them dealing with stressful situation.

## Data Availability Statement

The raw data supporting the conclusions of this article will be made available by the authors, without undue reservation.

## Ethics Statement

The studies involving human participants were reviewed and approved by the Ethics Committee of the Institute of Psychology, the Chinese Academy of Sciences. Written informed consent for participation was not required for this study in accordance with the national legislation and the institutional requirements.

## Author Contributions

JF, JiL, ZM, and JuL designed the study. JF supervised the data collection, carrying out the statistical analysis, and wrote the paper. XL participated in the revision of the article and data analysis. JiL collected the data and assisted with writing the article. ZM was responsible for the collection of the data. JuL supervised the whole project and provided critical comments and suggestions in writing and revising the article. All authors have approved the submitted version for publication.

## Funding

This work was supported by National Key Research and Development Program of China (2018YFC2000300, 2018YFC2001701, 2020YFC2003000, 2016YFC1305900, 2017YFB1401203) and the National Natural Science Foundation of China (32071079, 31861133011, 31671157, 31711530157).

## Conflict of Interest

The authors declare that the research was conducted in the absence of any commercial or financial relationships that could be construed as a potential conflict of interest.

## Publisher’s Note

All claims expressed in this article are solely those of the authors and do not necessarily represent those of their affiliated organizations, or those of the publisher, the editors and the reviewers. Any product that may be evaluated in this article, or claim that may be made by its manufacturer, is not guaranteed or endorsed by the publisher.

## Footnotes

^1^Using this inclusion criteria, about 18.4% were identified as older adults with HRCI from the screening sample of over 2,000 older adults in the communities, which was roughly the same as the proportion identified in previous studies (about 20%; Ecay-Torres et al., 2018; Chosy et al., 2019).

^2^The satisfaction score had no effects on any of the dependent variables, such as changes of emotional states or adoption of protective behaviors. Therefore, this variable was not included in further data analysis.
